# Tomotherapy: Comparison of Hi-ART, Tomo-HD, and Radixact

**DOI:** 10.7759/cureus.30949

**Published:** 2022-10-31

**Authors:** Hiromasa Kurosaki, Kenta Hirayama, Masaki Takahashi, Masahiro Uematsu, Etsuko Tate

**Affiliations:** 1 Department of Radiology and Radiation Oncology, Edogawa Hospital, Tokyo, JPN

**Keywords:** irradiation time, radixact, intensity modulated radiotherapy, rectal cancer, tomotherapy

## Abstract

Aim

In this study, we compared three generations of tomotherapy (Hi-ART, Tomo-HD, and Radixact). This is to study the difference among tomotherapy systems in terms of dose distribution to planning target volume and organs at risk, and irradiation time.

Materials and methods

The treatment planning CT and contour information used were seven cases of rectum cancer pre-operative irradiation. The contour information used was the planning target volume, and the organs at risk were set as the bladder and body. Optimization was conducted at each planning station using the parameters that were actually used in a clinical setting. The prescribed radiation dose was 25 Gy in five fractions and normalized at the isodose line, covering 95% of the planning target volume.

Results

There were no significant differences in planning target volume among the three models. Meanwhile, Hi-ART had a significantly higher dose than Tomo-HD and Radixact at body D_50%_. Radixact shortened the irradiation time by approximately 15% compared to Hi-ART/Tomo-HD.

Conclusion

Planning target volume dose distribution of tomotherapy devices was not different. Radixact required a significantly shorter time than Hi-ART and Tomo-HD.

## Introduction

TomoTherapy® (Accuray Inc., Sunnyvale, California) is a dedicated device for helical intensity-modulated radiotherapy (IMRT) that allows image guidance using megavolt-computed tomography (MV-CT). Several hardware and software modifications have been implemented since its clinical launch in 2002. The first TomoTherapy system was called Hi-ART, a dedicated helical IMRT device. The second-generation system, Tomo-HD, enabled fixed IMRT along with helical IMRT using TomoDirect™. Furthermore, the introduction of TomoEDGE™ allowed changes in jaw width at the time of dose irradiation by moving a dynamic wedge. These systems have been integrated into third-generation Tomo-HDA.

The fourth-generation system, Radixact®, the current and latest model, has both a redesigned gantry and treatment-planning system. Radixact® showed that kilovolt-computed tomography (KV-CT) using fast ClearRT™ (Accuray Inc., Sunnyvale, California) significantly improved image-guided image quality and imaging speed compared with MV-CT [1]. Furthermore, treatment with a high dose rate of 1000 cGy/min was possible compared with 850 cGy/min for Hi-ART, Tomo-HD, and Tomo-HDA. The Precision® treatment planning system has also been redesigned.

To date, only a few studies have compared these TomoTherapy generations [2-4]. Kraus et al. retrospectively investigated Hi-ART and Radixact from various perspectives [3]. They reported that the irradiation time was reduced by approximately 30% with Radixact compared to that with Hi-ART. However, the same cases were not used, and the modulation factor (MF), which affects the irradiation time, was calculated differently. Furthermore, they did not compare dose parameters. Therefore, it cannot be concluded that Radixact is superior to Hi-ART in terms of irradiation time based on these findings.

Therefore, in the present study, we compared dose parameters and irradiation time for Hi-ART, Tomo-HD, and Radixact using rectal cancer pre-operative irradiation as a model.

## Materials and methods

Using radiotherapy devices 

The radiotherapy devices used were Hi-ART, Tomo-HD, and Radixact (all devices were from Accuray Inc., Sunnyvale, California) (Table 1). Tomo-HD and Radixact were operated using the TomoEDGE.

**Table 1:** Differences in the three generations of tomotherapy used in this study -: Equipped without TomoEDGE +: Equipped with TomoEDGE

	Hi-ART	Tomo-HD	Radixact
TomoEDGE	-	+	+
Dose rate (cGy/min)	850	850	1000
Planning station (version)	Hi-ART planning station (5.1.1.6)	Tomo-HD planning station (5.1.1.6)	Accuray precision (3.3.1.2)

Contouring and treatment planning

Seven rectal cancer cases of preoperative irradiation were used for treatment-planning CT and contour information from April to May 2022 at the department of radiology and radiation oncology of Edogawa Hospital. The contour information used was the planning target volume (PTV), and the organs at risk (OAR) were the bladder and body (Table 2).

**Table 2:** Parameters and volume used in this study PTV = planning target volume

Parameters	Average volume (ml)	Volume range (ml)
PTV	947	750 - 1328
Bladder	108	34 - 283
Body	27792	19980 - 35752

Optimization was conducted at each planning station using parameters that were actually used in a clinical setting. All plans were calculated with helical IMRT with a 5-cm jaw. The prescribed radiation dose was 25 Gy in five fractions and was normalized at the isodose line covering 95% of the PTV. The modulation factor (MF) was 1.75-2.2 (1.8 in four out of seven cases); the pitch used was 0.231 in five cases and 0.303 in two cases. The evaluation parameters were as follows: PTV: D_98%_, D_50%_, D_2%_; bladder: D_98%_, D_50%_, D_2%_; body: D_50%_; and irradiation time. The dose parameters were calculated using the RayStation treatment planning system (Ray Research Laboratories, Stockholm, Sweden).

Statistical analysis

Excel 2019 (Microsoft, Redmond, Washington) was used for statistical analysis. One-way analysis of variance was used for tests, and when a significant difference was observed, Bonferroni's multiple comparison test was conducted with p<0.05 set as a significant difference.

## Results

Table 3 shows the results. There were no significant differences in the PTV among the three models. At a body D_50%_, Hi-ART had a significantly higher dose than Tomo-HD and Radixact.

**Table 3:** One-way analysis of variance was used for tests, and when a significant difference was found, Bonferroni's multiple comparison test was conducted *Hi-ART vs Tomo-HD: p=0.037, Hi-ART vs Radixact: p=0.036 **Hi-ART vs Radixact: p<0.001, Tomo-HD vs Radixact: p=0.008 PTV = planning target volume

Structure		Hi-ART	Tomo-HD	Radixact	p-value
PTV	D_98%_	24.46±0.13	24.45±0.13	24.51±0.13	0.6
	D_50%_	25.68±0.14	25.68±0.16	25.68±0.16	0.99
	D_2%_	26.13±0.18	26.13±0.16	26.04±0.23	0.99
Bladder	D_98%_	7.41±2.04	7.41±2.11	7.09±1.92	0.93
	D_50%_	13.14±2.83	13.10±2.97	13.15±2.86	0.98
	D_2%_	23.90±1.15	23.80±1.09	23.51±1.10	0.83
Body	D_50%_	2.15±1.39	0.85±0.35	0.85±0.36	0.007*
Irradiation time	(second）	530.5±50.0	512.2±73.5	441.9±52.3	0.029**

**Figure 1:**
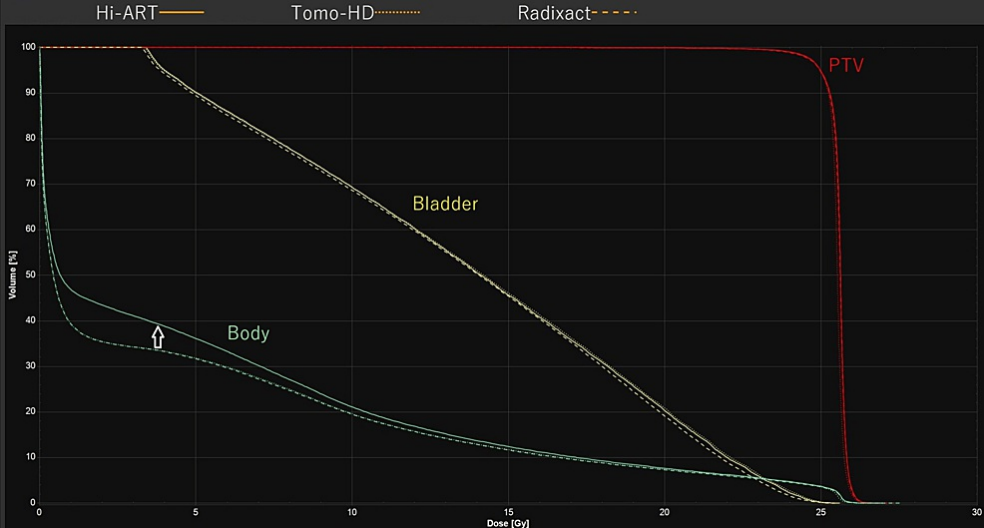
Representative example of a dose-volume histogram PTV = planning target volume

Figure 1 shows a representative example of a dose-volume histogram. On the dose-volume histogram (DVH), there is no difference among the three systems for dose distribution to planning target volume and bladder, respectively. For body, there is no significant change seen between Tomo-HD and Radixact, while a notable difference is seen between Hi-ART and the other two systems (indicated by an arrow).

There were no differences between Hi-ART and Tomo-HD in terms of irradiation time. Radixact® required a significantly shorter time than Hi-ART and Tomo-HD (Hi-ART: 530.5 seconds, Tomo-HD: 512.2 seconds and Radixact (441.9 seconds).

## Discussion

Radixact not only improves the image quality of MV-CT, but it also incorporates image guidance with KV-CT for improved throughput [2,3,5]. Tegtmeier et al. reported that KV-CT required only 25-45 seconds, whereas MV-CT required two to five minutes [1]. Our investigation showed no difference in the PTV or OAR (the bladder) between Hi-ART, Tomo-HD, and Radixact. There was no difference in body D_50%_. In other words, there was no improvement in dose distribution between Tomo-HD and Radixact. Additionally, the presence or absence of TomoEDGE had an impact on the body D_50%_ in Hi-ART [6,7]. Therefore, it is desirable to consider early replacement with Radixact.

Kraus et al. reported that the average irradiation time of craniospinal axis irradiation was 915.9 seconds; however, Radixact shortened the irradiation time by approximately 30% to 636.2 seconds [3]. However, this study did not use the same cases; therefore, Radixact might have allowed for better planning than Tomo-HD based on clinical experience. In fact, they changed the pitch (Hi-ART: 0.3, Radixact: 0.441) and used a lower MF for Radixact (Hi-ART: 2.4, Radixact: 2.3).

MF and jaw size have a large impact on irradiation time in tomotherapy. Kerf et al. found that MF did not affect the treatment time when the pitch was small (≤0.20) when planning for oropharyngeal cancer. In contrast, when the pitch was large (≥0.25), lowering the MF until the gantry speed was maximized reduced both treatment time and plan quality [8]. Furthermore, Ishibashi et al. reported that MF reduction from 3.0 to 1.8 in hippocampus-sparing whole-brain irradiation resulted in almost no deterioration in plan quality and a shorter irradiation time [9]. The irradiation time at an MF of 1.8 was approximately 60% of that at an MF of 3.0, and the irradiation time decreased linearly during this period. In the present study, the irradiation time in Radixact showed a significant decrease of 15% compared with Hi-ART or Tomo-HD; however, this was almost consistent with the increase in dose rate. Therefore, these results suggest that the reduced irradiation time in Radixact was due to the increased dose rate, and this result differed from that reported by Kraus et al. In the future, this should be investigated in other diseases.

Yokoyama et al. compared TomoTherapy with Halcyon™ (Varian Medical Systems, Helsinki, Finland), a CT-shaped radiotherapy device, and showed that TomoTherapy was superior in terms of dose quality; however, Halcyon was superior in terms of irradiation time [10]. Long irradiation time has been reported as a problematic aspect of TomoTherapy. Radixact has a significantly shorter irradiation time than Hi-ART and Tomo-HD, resulting in improved throughput. Furthermore, Radixact has a new respiratory gating system called Synchrony® [11,12]. A limitation of our study is that we examined a small number of cases using preoperative irradiation of rectal cancer as a model. Although there was no significant difference in dose distribution between Radixact and Tomo-HD, there were advantages in terms of image guidance by the respiratory gating system and KV-CT, which will inform the upgrade of the devices.

A limitation of this study is that only preoperative rectal cancer irradiation was used as a model. Further studies are needed for small, nearly spherical PTVs, such as irradiation for prostate cancer, and for complex, larger PTV volumes, such as hippocampus-sparing whole-brain irradiation.

## Conclusions

In this study, we compared dose parameters and irradiation time for Hi-ART, Tomo-HD, and Radixact using rectal cancer pre-operative irradiation as a model. Planning target volume dose distribution of TomoTherapy devices was not different. Radixact required a significantly shorter time than Hi-ART and Tomo-HD. Tomotherapy has evolved into Hi-ART, Tomo-HD, and Radixact in many aspects.
